# Prevention of Natural Disaster-Induced Cardiovascular Diseases

**DOI:** 10.3390/jcm13041004

**Published:** 2024-02-09

**Authors:** Minako Yamaoka-Tojo, Taiki Tojo

**Affiliations:** 1 Department of Rehabilitation, Kitasato University School of Allied Health Sciences, Sagamihara 252-0373, Japan; 2 Department of Cardiovascular Medicine, Kitasato University Graduate School of Medical Sciences, Sagamihara 252-0373, Japan; 3 Department of Cardiovascular Medicine, Kitasato University School of Medicine, Sagamihara 252-0374, Japan

**Keywords:** preventive cardiology, stress, hypertension, heart failure, pulmonary thromboembolism, Takotsubo cardiomyopathy, venous thrombosis

## Abstract

Natural disasters, such as floods and landslides caused by heavy rainfall, earthquakes, and tsunamis, can induce stress, which may contribute to the onset and aggravation of various cardiovascular diseases. The circulatory system is most susceptible to the effects of stress, and stress-related cardiovascular diseases, such as Takotsubo cardiomyopathy, pulmonary thromboembolism, hypertension, stroke triggered by increased blood pressure, and acute myocardial infarction, can occur during natural disasters. The risk of developing angina pectoris, arrhythmia, sudden cardiac death, and heart failure increases rapidly and can persist for several months. Moreover, treating cardiovascular diseases is essential during the acute phase, and continuous disease management is necessary during the chronic phase. However, disaster medical care for the victims must be given priority during natural disasters, which may cause a delay in diagnosis or access to necessary treatment for pre-existing medical conditions that could worsen or may cause death in patients with cardiovascular diseases. In this review, we summarize the predisposing factors for cardiovascular diseases that have been obtained through disasters such as major earthquakes and provide potential insights to help medical staff prevent the onset and aggravation of cardiovascular diseases during disasters.

## 1. Introduction

Stress plays a critical role in the onset and exacerbation of various cardiovascular diseases [1]. The circulatory system is most susceptible to the effects of stress. In particular, disasters such as earthquakes and floods aggravate stress-related cardiovascular diseases, such as Takotsubo cardiomyopathy [2], pulmonary thromboembolism [3], hypertension [4], and increased blood pressure. The risks of developing stroke [5], acute myocardial infarction/angina pectoris [6,7,8,9], arrhythmia [8], sudden cardiac death [9], and heart failure [6] increase rapidly and persist for several months. Table 1 summarizes the common cardiovascular diseases that are likely to occur during a disaster’s acute and chronic stages and their appropriate responses.

Using national population data, researchers assessed the link between the Great East Japan Earthquake and out-of-hospital cardiac arrests presumed to be of cardiac origin [10]. Among 6733 adults in the affected region, the risk of such cardiac arrests significantly increased in the first (1.70), second (1.48), third (1.47), and fourth (1.26) weeks post-earthquake. Notably, there was no significant increase in risk among men aged 18–74, but a significant increase was observed among women in the same age group during the initial two weeks after the earthquake.

However, treating cardiovascular diseases is important in the acute phase, and continuous disease management is necessary in the chronic phase. Moreover, priority must be given to disaster medical care to provide the minimum necessary medical care to as many victims as possible. Consequently, several pre-existing medical conditions may worsen owing to secondary factors, such as delay in diagnosis or access to appropriate treatment. In the case of cardiovascular diseases, failure to receive prompt treatment can sometimes lead to death.

## 2. Risk of Onset and Exacerbation of Cardiovascular Disease in Disaster-Stricken Areas

During the initial three days following a large-scale earthquake, the majority of fatalities result from direct health impacts induced by the disaster, such as crush injuries or drowning. However, cardiovascular disease and disaster-related deaths, such as those from infectious diseases and mental illnesses, will increase after the fourth day and several weeks after people are relocated to evacuation centers [11]. To prevent cardiovascular disease, we need to prevent increased blood pressure and arrhythmia due to stress and insomnia, worsening of the condition due to insufficient disease management due to the inability to take regular medications, consumption of foods with high salt content, and emergency foods high in fat and carbohydrates. Furthermore, unbalanced eating habits, in which people are forced to eat under difficult conditions, may increase the risk of cardiovascular events.

**Figure 1:**
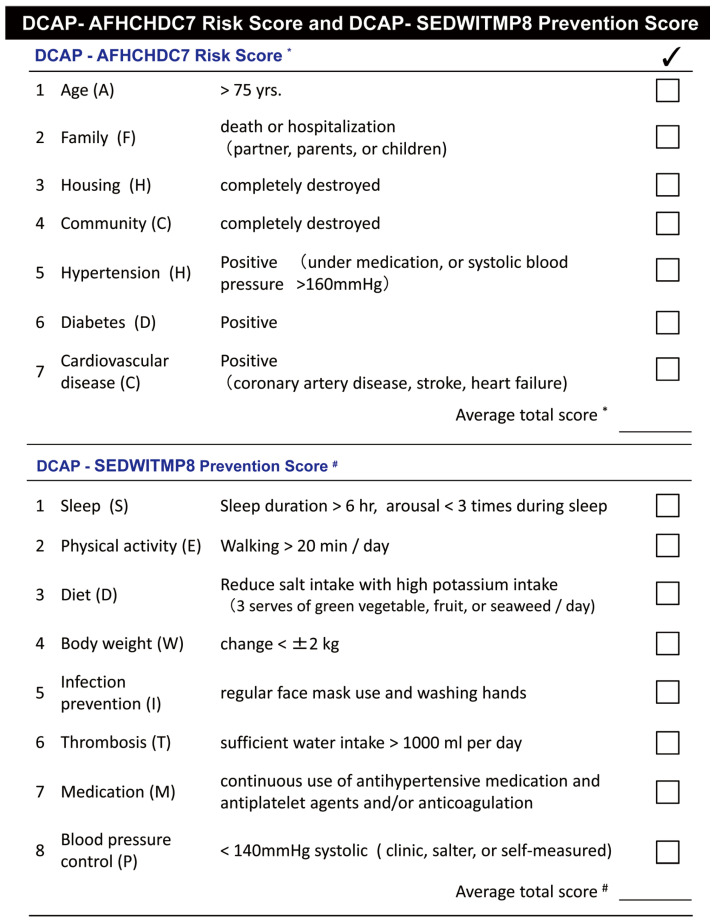
Disaster cardiovascular prevention (DCAP) risk score (AFHCHDC7) and prevention score (SEDWITMP8). * Total number of each risk factor as the individual risk score (0–7 points). The individual with 4 points or more is in the high-risk group. # Total number of each prevention factor as the individual prevention score (0–8 points). Target prevention score is 6 or more, particularly in high-risk patients [12].

The “disaster cardiovascular prevention risk score/prevention score”, which includes seven items, is effective for stratifying the risk of developing and exacerbating cardiovascular disease in disaster-stricken areas (Figure 1) [11,12,13]. The risk of disaster-related death is higher if four of the seven items are applicable to participants. Therefore, receiving lifestyle guidance and generous support from medical professionals and specialists at an early stage is essential. This table is useful for stratifying the risk of developing and exacerbating cardiovascular diseases during disasters. Disaster victims will be given a disaster cardiovascular prevention score (Figure 1) and provided with more specific guidance so they can complete as many items as possible. 

## 3. Essential Precautions to Prevent Cardiovascular Disease during Disasters

The lifestyle restrictions and excessive stress faced by residents in disaster-stricken areas pose major health risks. Owing to the changes in the environment and mental stress caused by disasters, individuals are susceptible to illnesses, often resulting in physical and mental problems such as headaches, dizziness, diarrhea, constipation, and insomnia due to anxiety. 

Regular preventive measures help prevent cardiovascular diseases, especially during natural disasters. Comprehensive risk management is fundamental and includes ensuring adequate sleep, continuing physical activity and exercise, maintaining a good diet and weight, continuing to take oral medications, controlling blood pressure, and quitting smoking [14]. Moreover, drinking sufficient water is necessary. Unless there are established fluid restrictions owing to heart failure or kidney disease, 1 L of fluid per day is recommended to prevent thrombosis. In particular, smokers have a high risk of developing thrombosis; therefore, they should be encouraged to quit smoking during disasters.

The specific content of the guidelines regarding cardiovascular disease prevention measures in disaster-stricken areas is presented in this section.

### 3.1. Preventing Venous Thrombosis While Living in an Evacuation Center or Evacuating in a Car

Prolonged periods of reduced physical activity, dehydration, and leg injuries are associated with a high risk of venous thrombosis. Instructions to prevent the onset of symptoms include: (1) avoid sleeping while sitting in a car seat for long periods, (2) frequent exercise for ankles and massaging the calves, (3) stay hydrated and avoid using the toilet, and (4) if possible, use cots at evacuation centers [15].

Alerts and educational activities to prevent thrombosis while living in evacuation centers, such as active exercise, walking, and avoiding long periods of immobility, are important measures to prevent cardiovascular diseases during disasters [16]. The Japanese Society of Cardiology has published the “Heart Disease Prevention Awareness Poster for Disaster Victims” as an awareness campaign to prevent economy class syndrome [17], which often occurs during disasters. Using elastic stockings with proper guidance can increase the effectiveness of preventing venous thrombosis and pulmonary thromboembolism in situations that necessitate sleeping in a car or the inability to exercise in an evacuation center.

### 3.2. Measures against Disaster Hypertension

Severe stress and discontinuation of antihypertensive drugs during disasters can result in disaster hypertension, which can cause plaque rupture and arterial thrombosis, leading to acute myocardial infarction, heart failure, cerebral hemorrhage, cerebral infarction, deep vein thrombosis, pulmonary thromboembolism, and sudden death. Hypertension triggers the development of various cardiovascular diseases [18]; therefore, efficient measures must be taken to monitor blood pressure as early as possible, and a system will be established to prevent cardiovascular disease in the event of a disaster, such that people with a systolic blood pressure of ≥140 mmHg or higher receive medical attention. 

Natural disasters, including earthquakes, hurricanes, large-scale wildfires, and floods, can each give rise to distinct stressors. Depending on the severity and circumstances of the disaster, the effects on blood pressure elevation are likely to vary. Particularly noteworthy is the potential for chronic blood pressure elevation, especially during prolonged evacuations, posing a heightened risk of accelerating arteriosclerosis and developing cardiovascular diseases. Systolic blood pressure increases by an average of 5–25 mmHg over the 2–4 weeks after an earthquake [12,19]. However, the increase in blood pressure after a disaster is usually transient and often subsides after the fourth week of the disaster [11]. However, caution is required in patients with increased salt sensitivity, such as older adults and those with chronic kidney disease, obesity, and metabolic syndrome, which may prolong the elevated blood pressure [19]. Extended stays at evacuation centers after major disasters can exacerbate hypertension due to factors like excessive salt intake, stress, sleep disorders, increased alcohol and unhealthy food consumption, and reduced physical activity. Considering these factors, comprehensive guidance and countermeasures are essential to prevent cardiovascular diseases in disaster-stricken areas, with the effectiveness of multidisciplinary interventions.

### 3.3. Weight Management during Disasters

In addition to blood pressure management, weight management is critical to prevent cardiovascular diseases. Dehydration or malnutrition was suspected if an individual had lost ≥2 kg of body weight before the disaster, and measures are to be taken to prevent the onset of arrhythmias, such as atrial fibrillation, and serious diseases, such as thrombosis. Conversely, an increase in body weight of ≥2 kg or the presence of swelling warrants necessary checks to detect the onset of heart failure, acute exacerbation of chronic heart failure, or worsening of chronic kidney disease. Especially in older adults, heart failure often worsens after a disaster. Therefore, if a person experiences symptoms such as nocturnal dyspnea or loss of appetite accompanied by weight gain, they should consult a doctor.

Excessive calorie intake and a lack of exercise can lead to obesity due to continuous weight gain in individuals living in a shelter for a long time. Sudden weight gain requires attention as it increases the risk of developing arteriosclerotic diseases, such as increased blood pressure, abnormal glycolipid metabolism, and a further decrease in physical activity due to joint pain caused by weight gain.

### 3.4. Keep Takotsubo Cardiomyopathy in Mind

“Takotsubo cardiomyopathy” is a condition in which the heart’s contractile function decreases, and pump failure occurs after experiencing excessive mental stress due to strong pain stimulation or a large-scale disaster. This may be because of the disruption in the autonomic nervous system due to sudden stress. Because the patient experiences symptoms similar to those of acute myocardial infarction, such as chest pain, chest tightness, and difficulty breathing, the patient must be transported to a medical facility that can provide cardiovascular treatment. It was particularly common among middle-aged and older women during the Great Hanshin–Awaji Earthquake and Niigata–Chuetsu Earthquake [18].

### 3.5. Continued Treatment to Prevent the Worsening of Cardiovascular Disease

Continuous oral medication is crucial for managing cardiovascular disease, and its discontinuation during disasters can worsen the condition. In case of evacuation, people should be instructed to carry essential oral medications, but challenges may arise in accessing prescription drugs depending on the crisis. A post-evacuation system should be established for individuals to consult medical professionals for necessary guidance once the crisis subsides.

During a disaster, oral medication is likely to become irregular; therefore, special care must be taken to ensure non-discontinuation of essential medications in patients such as those (1) on warfarin for artificial valve surgery, (2) with a coronary artery stent, (3) on three or more types of medications for high blood pressure, or (4) with heart failure. Patients receiving medical treatment must consult a physician as soon as possible.

In the event of a large-scale disaster affecting a vast geographical area, the possibility of disruption in transportation infrastructure and the distribution of pharmaceuticals should be anticipated in advance. In preparation for emergencies, the mutual storage of medical data, such as drug information, medical history, and disease management status, among medical institutions is considered important as part of the efforts to raise awareness regarding the use of information and communication technology (ICT). In addition, it is necessary to maintain a regular stock of prescription drugs, replenish drugs from pharmaceutical manufacturers and wholesalers, have logistics backup systems with ICT, and have patient support systems in back-office hospitals [18]. 

When providing medical support in disaster-stricken areas, prioritize self-sufficiency in necessary items. Appointing a disaster medical coordinator can enhance efficiency, ensuring adherence to instructions and seamless information sharing among medical teams. Collecting detailed information from the disaster area, delivering locally tailored medical care, and promptly reporting findings are crucial. Establishing a network for medical cooperation and support activities, applicable in both regular times and during disasters, is highly recommended.

### 3.6. How to Prevent Sudden Death in Disaster-Stricken Areas

Many acute cardiovascular diseases occur following major earthquakes, and simultaneously, the number of cardiac arrests and sudden deaths increases [18]. A multidisciplinary approach is often warranted at disaster sites to conduct regular discussions on measures to prevent the sudden deaths of disaster victims. In addition to preventing the occurrence of diseases that cause sudden deaths during disasters, necessary steps to prepare living environments, educate disaster victims, prepare medical equipment, and provide drug treatments in response to the occurrence of these diseases are essential.

Sudden deaths do not immediately increase after a large-scale disaster, and the diseases that cause sudden deaths vary depending on the time of the disaster. Hypothermia is the cause of cardiac arrest within 24 h, whereas acute myocardial infarction, Takotsubo cardiomyopathy, ventricular arrhythmia, acute heart failure, acute aortic dissection, worsening of renal failure, and hypothermia are the causes of cardiac arrest within 24–72 h On days 4 to 7, cardiac arrests due to acute myocardial infarction, acute heart failure, pulmonary thromboembolism, stroke, or worsening of renal failure are common, and cardiac arrests reported after day 7 may be frequently associated with Takotsubo cardiomyopathy. Regarding the balance between medical demand and supply, the supply is anticipated to increase four days after a disaster. Factors such as regional disparities, delays in patient discovery, and poor supply due to a lack of information exchange must be fully resolved to implement appropriate disaster medical care [18].

## 4. Cardiovascular Disease Prevention in Evacuation Centers

Establishing a medical team centered on doctors and medical staff at evacuation centers is imperative to support evacuees’ blood pressure management and continued treatment. As various facilities are used as evacuation centers during disasters, assessing and improving the living environment is desirable [18]. Specifically, maintaining sufficient living space for each evacuee is important (ideally ≥ 3.5 m^2^ per person). Confirming whether there are partitions for each household, whether there is a set time for lights out, and whether the room temperature inside the evacuation center is appropriate. Furthermore, the environmental conditions should be checked in each evacuation center as follows: Is wearing shoes acceptable? Are there sufficient lifelines for power generation, gas, and water supply? Are there communication methods such as mobile phones and satellite phones? Are there sufficient sanitary supplies such as masks, alcohol disinfectants, and tableware? In addition, health providers should evaluate whether sanitary environments, such as bathrooms and toilets, are sufficient and whether there are people who require care or are unwell.

Different medical teams are often in charge of medical activities in disaster-stricken areas, mainly in evacuation centers, and information sharing is vital for improving the efficiency of medical care. Disaster survivors carry their own medical records and present them to medical professionals during consultations so that patients can write down their medical history, medications, and symptoms, and doctors and medical staff can provide information on cardiovascular diseases. A record sheet for recording and leaving the evacuation center can be downloaded from the Japanese Society of Cardiology website [20].

In disaster-stricken areas, people are forced to live in evacuation centers in environments that are completely different from their daily lives. Therefore, medical staff provide life guidance that is feasible for disaster victims, even with limited medical resources.

### 4.1. Ensuring Sufficient Sleep and Stress Management

To help disaster victims in evacuation centers obtain adequate sleep, measures such as turning off lights at night, using eye masks and earplugs, and using mattresses to prevent vibrations are useful for improving the sleeping environment. People should strive to achieve at least 6 h of quality sleep.

Persistent insomnia, or if panic, excitement, or absentmindedness are strong, it is necessary to take steps to improve “mental care”, such as consulting a specialist at the earliest; however, immediate medical intervention is not recommended. In cases of extreme difficulty, it is important to remember that new anxiety, depression, irritation, and impatience that arise after a disaster can happen to anyone, and most of them are temporary. The patients should be informed that there are options to monitor the situation calmly, such as using a consultation room or telephone counseling, and confirm that there is a system where they can receive psychological support [21].

The functions of mental health and medical institutions in the affected area will temporarily decline during a major mass disaster, and new mental health problems will arise due to disaster-induced stress, increasing the demand for mental health care. Disaster Psychiatric Assistance Teams (DPATs) are organized in prefectures and ordinance-designated cities to provide specialized psychiatric care and support mental health insurance activities in the event of a disaster (https://www./dpat.jp/ (accessed on 20 January 2024)). Records of disaster response from individual business records should be made available for reference even in situations where the DPAT is not dispatched to a large-scale mass disaster so that the history of disaster support can be seen in medium- and long-term health activities [22].

Furthermore, numerous incidents that exacerbate stress, such as fighting over blankets, having items stolen, violence, and sexual assault, have been reported in disaster-stricken areas. Crisis management is effective in reducing crime through the cooperation of evacuees, such as warning and awareness campaigns inside evacuation centers to avoid becoming victims, night watchmen, and daytime patrols.

### 4.2. Increasing Physical Activity and Exercise

As for physical activity, one should try to walk for at least 20 min every day. Particular attention should be paid to the risk of developing deep vein thrombosis or pulmonary thromboembolism due to sitting in a car or a small evacuation center for long periods or remaining immobile for extended periods. Although it is necessary to prevent venous thrombosis in the lower limbs by exercising and walking regularly, an evacuation environment often induces physical inactivity.

### 4.3. Good-Quality Diet

Even while consuming emergency food, salt intake should be reduced, and potassium intake should be paid attention to. However, caution is required to restrict potassium intake in individuals with hyperkalemia, such as those with chronic kidney disease. Evacuation diets have a number of restrictions, but ideally, the diet should include plenty of potassium-rich foods such as green and yellow vegetables, fruits, seaweed, and unsalted vegetable juices.

For patients with high blood pressure or heart failure, the daily salt intake should be less than 6 g. Emergency foods are often high in salt, and individuals living in an evacuation center should be concerned about their salt intake by avoiding the consumption of high-salt foods, such as cup noodle soup or rice ball ingredients [15].

### 4.4. Infectious Disease Prevention

The basics for preventing infectious diseases include wearing masks, disinfecting hands with alcohol, washing hands, isolating patients suspected of having an infectious disease, establishing a treatment system to prevent clusters of infectious diseases, and implementing hygiene measures to prevent food poisoning during the rainy and summer seasons. Therefore, management strategies should be considered, and cooperation throughout the evacuation center is needed to prevent the spread of infectious diseases.

Preventive measures can be implemented at an early stage by obtaining information on the status of infectious disease outbreaks in nearby evacuation centers and other areas. However, the information on the infection status should be constantly updated at the respective evacuation centers. The information obtained should be aggregated and shared with local health authorities.

### 4.5. Continuation of Oral Medication, Blood Pressure Management, Smoking Cessation

Detailed information regarding blood pressure measurements and past disease management should be collected for patients who require regular prescriptions for antihypertensive drugs and anticoagulants, and a list of patients who require early medical intervention should be created. Doctors patrolling evacuation centers and nearby medical institutions are often unaware of the contents or dosage of medicines the patients are taking because they do not have medicine notebooks or cannot take their regular medicines with them during a disaster. A system through which patients can consult doctors should be built.

By controlling disaster hypertension, the onset of cardiovascular diseases in evacuation centers can be prevented. We aimed to create a situation in which blood pressure measurements could be taken at each evacuation center. Smoking not only increases blood pressure but also promotes blood clot formation, which can lead to stroke and acute myocardial infarction. Therefore, quitting smoking is an essential preventive measure for cardiovascular disease, even in evacuation centers.

For medical staff caring for patients with high blood pressure in disaster-stricken areas, it is reassuring to have a support system available for consultation at any time. The Japanese Society of Hypertension has set up a consultation desk for medical staff caring for patients with high blood pressure in disaster areas and evacuation centers, where they can ask questions about various issues related to high blood pressure. The queries can be sent via email (office@jpnsh.jp) or fax (03-6801-9787) to the Society Secretariat. The responses from the specialists may take some time. The name and contact information, such as phone number, reply addresses, fax, or email, should be provided to receive a response [20].

## 5. Cardiovascular Prevention in Disasters

As the saying goes, “disaster comes when you forget it”, but “preparation will prevent you from suffering disasters” is also important in preventing cardiovascular disease in the event of a disaster. People with chronic illnesses such as high blood pressure or cardiovascular disease should always keep a “stockpile” of medicines and a “medication notebook” with them in case of an emergency. A copy of the medication notebook can be placed in the wallets, and pictures of oral medications and drug information sheets can be stored in mobile phones for use during emergency situations. This is effective in providing countermeasure guidance that is as specific as possible, such as preparing for emergencies. The evacuation centers and related facilities need to be equipped with self-monitoring blood sugar monitors such as thermometers, weight scales, and blood pressure monitors.

Acquiring knowledge about prevention and educating patients about disease management is effective in preventing the onset and exacerbation of cardiovascular diseases. To prevent cardiovascular disease during disasters, medical personnel must be trained in understanding and promptly responding to specific cardiovascular disease prevention measures that are unique to disasters and improve the health literacy of the entire population regarding cardiovascular disease prevention, including during disasters. is desired. Gaining comprehensive knowledge and preparing for cardiovascular disease prevention in the event of a disaster and securing human resources who can provide leadership from the perspective of cardiovascular disease prevention is imperative, especially during emergencies.

## 6. Conclusions

Enhancing knowledge acquisition among healthcare providers and prioritizing patient education emerge as effective strategies for preventing and managing cardiovascular diseases during disasters. Moreover, the critical imperative is to secure proficient human resources capable of providing leadership, particularly focusing on preventive measures against cardiovascular diseases—an essential requirement, especially in emergencies. Recognizing the multifaceted nature of disaster management, fostering a robust network of well-informed professionals, and disseminating preventive measures to safeguard cardiovascular health during crises is crucial.

## Figures and Tables

**Table 1:** Common cardiovascular diseases associated with natural disasters.

Phase		Cardiovascular Diseases	Approach	Treatment
	Acute	Hypertension	Check history of hypertension	Reduce salt intake
			BP measurement	Adequate sleep
			BP monitoring	Antihypertensive medication
		Acute myocardial infarction	Transfer to a hospital that can provide life-saving treatment	Cardiac catheterization
				Coronary artery bypass surgery
		Aortic dissection	Transfer to a hospital that can provide life-saving treatment	Aortic replacement
			Strict BP control	Stent graft insertion
		Arrhythmia	Check pulse	Antiarrhythmic medication
			Check for signs of heart failure	anticoagulants
		Acute heart failure Takotsubo cardiomyopathy Acute exacerbation of CHF	Check for history of CHF Check for BW gain/edema Shortness of breath/chest pain Transfer to a hospital (severe cases)	BP management Ensure regular medications for HF Add diuretics (if required)
		Stroke	Transfer to a hospital that can provide life-saving treatment	Thrombolytic therapy
				Catheter intervention/surgery
		Venous thrombosis	Prevent dehydration Increase physical activity Elastic bandages/stockings	Anticoagulant therapy
Sub-acute		Pulmonary thromboembolism	Transfer to a hospital	Thrombolysis/removal by catheter or surgery

BP: blood pressure; BW: body weight; CHF: chronic heart failure; HF: heart failure.
